# *AtSIG6* and other members of the sigma gene family jointly but differentially determine plastid target gene expression in *Arabidopsis thaliana*

**DOI:** 10.3389/fpls.2014.00667

**Published:** 2014-11-25

**Authors:** Sylvia Bock, Jennifer Ortelt, Gerhard Link

**Affiliations:** Department of Biology and Biotechnology, University of BochumBochum, Germany

**Keywords:** chloroplast transcription, plant sigma factors, nuclear gene family, knockout mutants, RNA interference, plastid target gene expression

## Abstract

Plants contain a nuclear gene family for plastid sigma factors, i.e., proteins that associate with the “bacterial-type” organellar RNA polymerase and confer the ability for correct promoter binding and transcription initiation. Questions that are still unresolved relate to the “division of labor” among members of the sigma family, both in terms of their range of target genes and their temporal and spatial activity during development. Clues to the *in vivo* role of individual sigma genes have mainly come from studies of sigma knockout lines. Despite its obvious strengths, however, this strategy does not necessarily trace-down causal relationships between mutant phenotype and a single sigma gene, if other family members act in a redundant and/or compensatory manner. We made efforts to reduce the complexity by genetic crosses of Arabidopsis single mutants (with focus on a chlorophyll-deficient *sig6* line) to generate double knockout lines. The latter typically had a similar visible phenotype as the parental lines, but tended to be more strongly affected in the transcript patterns of both plastid and sigma genes. Because triple mutants were lethal under our growth conditions, we exploited a strategy of transformation of single and double mutants with RNAi constructs that contained sequences from the unconserved sigma region (UCR). These RNAi/knockout lines phenotypically resembled their parental lines, but were even more strongly affected in their plastid transcript patterns. Expression patterns of sigma genes revealed both similarities and differences compared to the parental lines, with transcripts at reduced or unchanged amounts and others that were found to be present in higher (perhaps compensatory) amounts. Together, our results reveal considerable flexibility of gene activity at the levels of both sigma and plastid gene expression. A (still viable) “basal state” seems to be reached, if 2–3 of the 6 Arabidopsis sigma genes are functionally compromised.

## Introduction

Despite the small number of genes in the chloroplast genome (Sugiura, 1992), plastid transcription is a surprisingly complex process. It involves two different RNA polymerases commonly named NEP (nuclear-encoded polymerase) and PEP (plastid-encoded polymerase) (Hedtke et al., 1997; Maliga, 1998). The latter is surrounded by multiple transcription factors (Shiina et al., 2005), important representatives of which are the nuclear-encoded sigma factors. Like their bacterial counterparts (Ishihama, 1988; Burgess and Anthony, 2001), these plant factors are thought to direct the (PEP) polymerase complex to its cognate promoters and ensure faithful transcription initiation. Again, as is the case in bacteria, the plastids of higher plants typically contain more than a single sigma factor species (e.g., a family comprising six proteins ATSIG1 - 6 in *Arabidopsis thaliana*) (Isono et al., 1997; Tanaka et al., 1997; Fujiwara et al., 2000; Shiina et al., 2009). This obvious analogy has therefore stimulated research addressing the role of individual members of the plant sigma factor family.

Work carried out with Arabidopsis knockout lines containing T-DNA insertions in single sigma genes has provided an initial picture, demonstrating the presence or absence of a recognizable mutant phenotype depending on the affected sigma gene as well as the developmental stage investigated (Tsunoyama et al., 2002; Hanaoka et al., 2003; Privat et al., 2003; Nagashima et al., 2004; Favory et al., 2005; Ishizaki et al., 2005; Loschelder et al., 2006; Schweer et al., 2006, 2009; Zghidi et al., 2007). A readily noticeable phenotype is evident for instance in the case of AtSIG6, where mutant lines tend to have strong chlorophyll deficiency and altered plastid target gene expression patterns, yet only in seedlings but not, e.g., in plants during the subsequent rosette leaf stages (Ishizaki et al., 2005; Loschelder et al., 2006; Schweer et al., 2006, 2009).

Unlike the situation in bacteria (Ishihama, 1988), none of the plastid sigma factors seems to have a “primary” essential role in a sense that its loss would confer a lethal or seriously compromised phenotype (Ortelt and Link, 2014). What then might be the reasons for the variable (non-lethal) phenotypes noticeable in plant sigma knockout lines? A perhaps most direct explanation would be that the plastid factors function in a partially overlapping manner, yet in a highly flexible way at different developmental stages, in different organs, and under variable environmental conditions. Clues supporting this idea come, e.g., from the modular architecture of the plant sigma factors, each of which has a C-terminal conserved region (CR) responsible for basal sigma activity and a N-terminal unconserved region (UCR) of regulatory function (Ortelt and Link, 2014).

Nevertheless, it seems likely that the current functional description of the underlying network is not yet complete. For instance, knocking out one single sigma gene may or may not have the consequence that a second (or third, fourth etc.) member of the factor family is functionally recruited in a specific developmental context. To reduce the complexity of the Arabidopsis sigma family, we took advantage of single and double mutant lines and also adopted RNAi (RNA interference) techniques (Fire et al., 1998) for that purpose. We then analyzed the gene expression situation (at RNA level) both for plastid target genes as well as for the members of sigma gene family themselves. We reasoned that such work could add novel information to help explain the flexibility of the plant sigma factor network and its phenotypic consequences.

## Materials and methods

### Arabidopsis mutant and RNAi lines, growth conditions, screening

Single mutant lines *sig1-2* (“*sig1*”), *sig3-2* (“*sig3*”), and *sig6-2* (“*sig6*”) in the Col-0 (ecotype Columbia) background of *Arabidopsis thaliana* were obtained from the GABI-Kat collection of T-DNA insertion lines (http://www.gabi-kat.de) (Rosso et al., 2003). Double knockouts *sig1 sig6* and *sig6 sig3* were generated from the single mutants by genetic crosses. Both single and double knockouts were transformed with sigma UCR sequences cloned into RNAi vector pHELLSGATE12 (Wesley et al., 2001) (www.csiro.au) using the Gateway system (Life Technologies). PCR-amplified sigma cDNA representing the full-size UCR (555 bp after the start codon for AtSIG1, 768 bp for AtSIG3, and 696 bp for AtSIG6) was inserted into the donor vector pDONR/zeo (Life Technologies) and mobilized to the destination vector as described in the Gateway manual (www.invitrogen.com). It was then introduced into Arabidopsis by Agrobacterium-mediated floral dip transformation (Clough and Bent, 1998) and progeny were screened using antibiotic selection, PCR and Southern blot analyses as described (Loschelder et al., 2006). The criteria for successful generation of double mutants, i.e., absence of an amplified PCR product using primers that flank the T-DNA insertion and presence of a product using a primer pair across the junction between T-DNA and sigma gene sequence, were tested for each candidate line (signal.salk.edu/tdnaprimers.2.html). Only those lines that fulfilled these requirements were further propagated and subsequent experiments were carried out with at least three independent T3 lines for each construct. Seeds were sown on MS medium (Murashige and Skoog, 1962) containing 0.4% (w/v) gelrite and 1% (w/v) sucrose, stratified at 4°C for 2 d, and then transferred to 24°C for germination and growth under short-day conditions (8 h light/16 h dark, 60 mmol m^−2^ s^−1^). Seedlings were harvested at day 6 or 10, or growth was continued until day 14, at which time plantlets were transferred to sterile soil for another 6 d until day 20. Tissue samples were immediately frozen in liquid nitrogen and stored at −85°C until use.

### Physiological parameters (chlorophyll content, root length measurements)

Chlorophyll content was measured using 8 replicates of 10 seedlings or young plantlets at each time-point (6 d, 10 d, 20 d after sowing) and measurements were repeated twice using independently grown plant material. Following weighing of seedlings, they were ground in 80% (v/v) acetone. The extract was centrifuged at 10,000 g for 10 min and the supernatant was used for photometric chlorophyll determination at 663 and 645 nm. For root lengths measurements, seedlings were grown as described above, yet using vertical positioning of Petri dishes. Length determination was carried out using ImageJ (http://imagej.nih.gov/ij) followed by graphical presentation. Values were means of three independent replicates each obtained from 20 seedlings.

### RT-qPCR detection of transcripts

Total seedling RNA (2 μg) was prepared and reverse-transcribed into cDNA as described by Loschelder et al. (2006), yet using oligo-(dT)-primers and the Moloney Murine Leukemia Virus Reverse Transcriptase (M-MLV; Promega). Quantitative real-time RT-PCR (RT-qPCR) was in an Illumina Eco System (www.illumina.com) with KAPA SybrFast qPCR MasterMix Universal (Peqlab) in a volume of 20 μl. Upon amplification (50°C for 2 min, 95°C for 10 min, followed by 40 cycles at 95°C for 10 s, 60°C for 30 s, and 72°C for 15 s), melting curve analysis was carried out, using the *Actin2 C*_t_ value as a reference for normalization. For each primer pair, the real-time experiments were carried out with at least three biological and technical replicate samples. Primers (Table 1) were selected on the basis of minimal sequence homology among the members of the Arabidopsis sigma gene family (Schweer et al., 2009).

**Table 1:** **Primers used for construction of RNAi lines, northern hybridization, and RT-qPCR**.

**Gene**	**Forward primer (5′ → 3′)**	**Reverse primer (5 ′ → 3 ′)**
**RNAi CONSTRUCTS**		
*SIG1*	GGGGACAAGTTTGTACAAAAAA GCAGGCTCCATGGCTACTGC AGCTGTTATAGGA	GGGGACCACTTTGTACAAGAAAG CTGGGTCCTACTTTCCACTAGAAA CATCAGAAAC
*SIG2*	GGGGACAAGTTTGTACAAAAA AGCAGGCTCCATGTCTTCTTGT CTTCTTCCTCAG	GGGGACCACTTTGTACAAGAAAG CTGGGTCCTAATTATGATCAACTT CCTGCGCAAC
*SIG4*	GGGGACAAGTTTGTACAAAAA AGCAGGCTCCATGGCGACGAC GATTCCCACTACA	GGGGACCACTTTGTACAAGAAAG CTGGGTCCTATCCAACAACAGGA ACACTAATTGT
**NORTHERN HYBRIDIZATION**		
*atpB*	GGGGAACCCGTTGATAATTT	AACGCTCAATTTTTCGTGCT
*rbcL*	TCGGTGGAGGAACTTTAGGC	TGCAAGATCACGTCCCTCAT
*RbcS*	TGGCTTCCTCTATGCTCTCTTC	GCTGAGGCGAGTTCACAAC
*rrn16*	CGGTATCTGGGGAATAAGC	GAAATTCCCTCTGCCCCTAC
*trnE*	CTACCCCCAGGGGAAGTC	AGGACTTGGTGATCTGCTACC
**RT-qPCR**		
*SIG1*	TTTTCTGCATGGTGGTTTGA	GTGGCACAGACAAATCGAGA
*SIG2*	TGTGCCCCTAAACACAACAA	GCTTTGCGACATCAAGCATA
*SIG3*	GTTGGGGGCTGCTGAGTTAT	TTCGGCGTAGTATCCACCAA
*SIG4*	CGTCTCCTCCTGTCCCTACA	TAGTGTCACCGCAAACCAGA
*SIG5*	AAGCCGATGATTCCAGCA	GAACACGAAGGAGCCGAAT
*SIG6*	CTGGAGAGGAGGCAGTTTGA	CCGGCAATTTCGTTTCAGATG
*Actin2*	CGCCATCCAAGCTGTTCT	TCACGTCCAGCAAGGTCA

### Northern blot hybridization

Total RNA was isolated as described by Chomczynski and Sacchi (1987). RNA (2 μg per lane) was fractionated, blotted, and hybridized, using gene-specific RNA probes (Table 1). The latter were generated by cloning of PCR-amplified gene segments into pGEM-T Easy (Promega), followed by *in vitro* transcription of constructs using T7 RNA polymerase (Promega) as described (Loschelder et al., 2006; Schweer et al., 2006). DIG (digoxigenin) labeling conditions and immunological detection using anti-digoxigenin antibody (Roche) were as detailed in the DIG user's manual (www.roche-applied-science.com).

## Results

### Characterization of sigma-deficient single and double mutants

**Figure 1:**
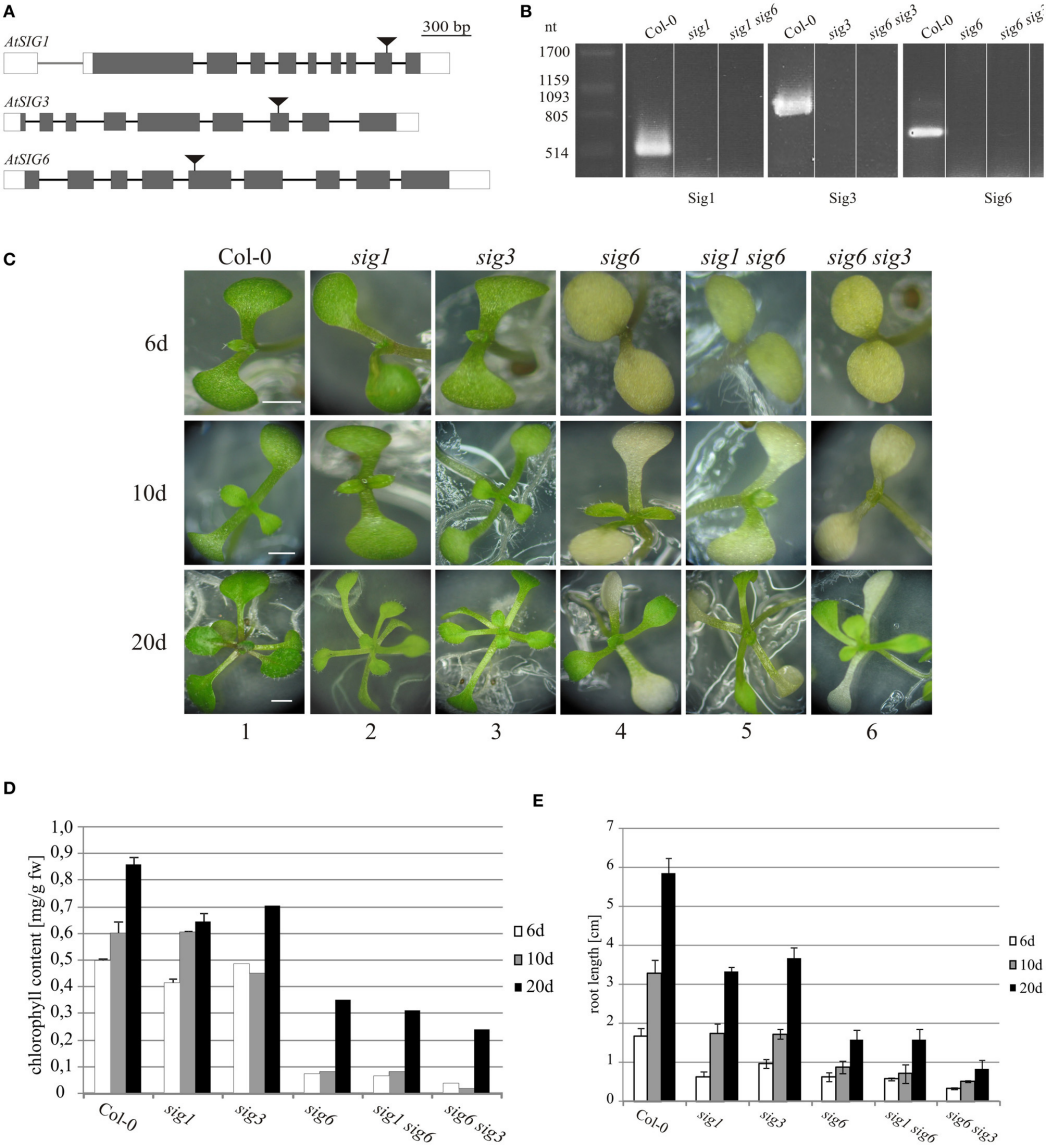
**Characterization of *Arabidopsis thaliana* sigma mutants. (A)** Exon/intron architecture of *AtSIG1* (At1G64860), *AtSIG3* (At3G53920), and *AtSIG6* (At2g36990). Regions colinear with the mature messenger RNA are boxed, including protein-coding (gray) and non-coding regions (white), while regions corresponding to introns of the RNA precursor are depicted as a single line. T-DNA insertion sites (in exon 9 of *AtSIG1*, exon 7 of *AtSIG3*, and exon 5 of *AtSIG6*, respectively) are marked by triangles. Genomic sequence, without T-DNA, is drawn to scale (scale bar on top). **(B)** RT-PCR detection of sigma factor transcripts in single mutant lines. RT-PCR detection of sigma factor transcripts in wildtype (Col-0) and mutant lines *sig1-2* (*SIG1*), *sig3-2* (*SIG3*), and *sig6-2* (*SIG6*). Total RNA was prepared from 10 d seedlings, reverse transcribed, and cDNA was amplified using gene-specific full-length primer pairs (Table 1) as described in “Materials and Methods.” **(C)** Visible phenotype. Arabidopsis wildtype (Col-0) as well as sigma single and double knockout lines were photographed at three different time-points during seedling and rosette leaf development (6, 10, and 20 d after sowing). Scale bars: 2 mm. **(D)** Chlorophyll content of 6 d, 10 d, and 20 d seedlings. **(E)** Root lengths measuments (for details, see “Materials and Methods”).

To raise information on the fine-tuning and complex regulation within the sigma gene family from Arabidopsis, we analyzed single and double sigma mutants. Starting material were the three single mutant lines *sig1-2, sig3-2*, and *sig6-2* defective in *AtSIG1, AtSIG3*, and *AtSIG6*, respectively (Figure 1A). These lines were then used in crosses to generate double mutants and selfed progeny lines were tested by genomic PCR for presence of the T-DNA insertion. As shown in Figure 1B, the amplification products detectable with wildtype DNA (using the primer pairs detailed in Table 1) were absent in either the single or double mutant lines, indicating a loss of sigma gene function in all these lines. This was confirmed by using primer pairs across the junction between T-DNA and sigma gene sequence, which generated amplification products (data not shown).

The single mutant lines *sig1-2* and *sig3-2* each have a visible phenotype resembling that of the wildtype (Figure 1C, panels 2 and 3). In contrast, the two double mutants *sig1 sig6* and *sig6 sig3*, each resulting from crosses with the *sig6* mutant line (panels 4), both reveal yellowish to white cotyledons, with only minimal light-green leaf primordia recognizable at day 10 (panels 5 and 6). Previous work (Ishizaki et al., 2005; Loschelder et al., 2006; Schweer et al., 2006) had shown that mutant alleles of the Arabidopsis sigma gene *AtSIG6* account for reduced chlorophyll content during seedling development. As is evident from Figure 1D, the *sig6-2* line (in comparison with the wildtype) reveals approximately 80% loss of chlorophyll at the 6 d and 10 d stages, respectively, but only 20% loss in 20 day old rosette plants. Chlorophyll quantification of the double mutants likewise shows highly reduced amounts, which (in *sig1 sig6*) are similar to or (in *sig6 sig3*) are even more pronounced than those of the *sig6* parental line at the 6 d and 10 d time-points. At day 20, there is an even further reduction by 15% in *sig1 sig6* and by almost 30% in *sig6 sig3* beyond the *sig6-2* level.

Root length (Figure 1E) is likewise reduced in the single mutants compared to wildtype, with a stronger effect noticeable for *sig6* than for *sig1-2* and *sig3-2*. The double mutant lines again show similar (*sig1 sig6*) or even greater (*sig6 sig3*) length reduction compared to *sig6-2*.

### Plastid gene expression at RNA level in wildtype, single and double knockout mutants

To assess consequences of sigma gene inactivation on plastid transcript patterns, we carried out northern blot analyses using total RNA from 6- and 10-day old single and double mutant lines. Since many plastid genes give rise to distinct (single or multiple) transcripts of relatively high abundance, this technique was selected to rapidly reveal stage-specific RNA expression patterns of organellar target genes of sigma-dependent transcription. Representatives of all three major classes of plastid genes, i.e., those for proteins (*atpB* and *rbcL*, the genes for the β subunit of the organellar ATP synthase and the large subunit of ribulose-1,5-bisphospate carboxylase-oxygenase or abbreviated Rubisco, respectively), ribosomal RNAs (*rrn16*, the gene for 16S rRNA) and transfer RNAs (*trnE*, the gene for glutamic acid-specific tRNA), were included in this analysis (for map position and sequence see www.ncbi.nlm.nih.gov/nuccore/7525012).

**Figure 2:**
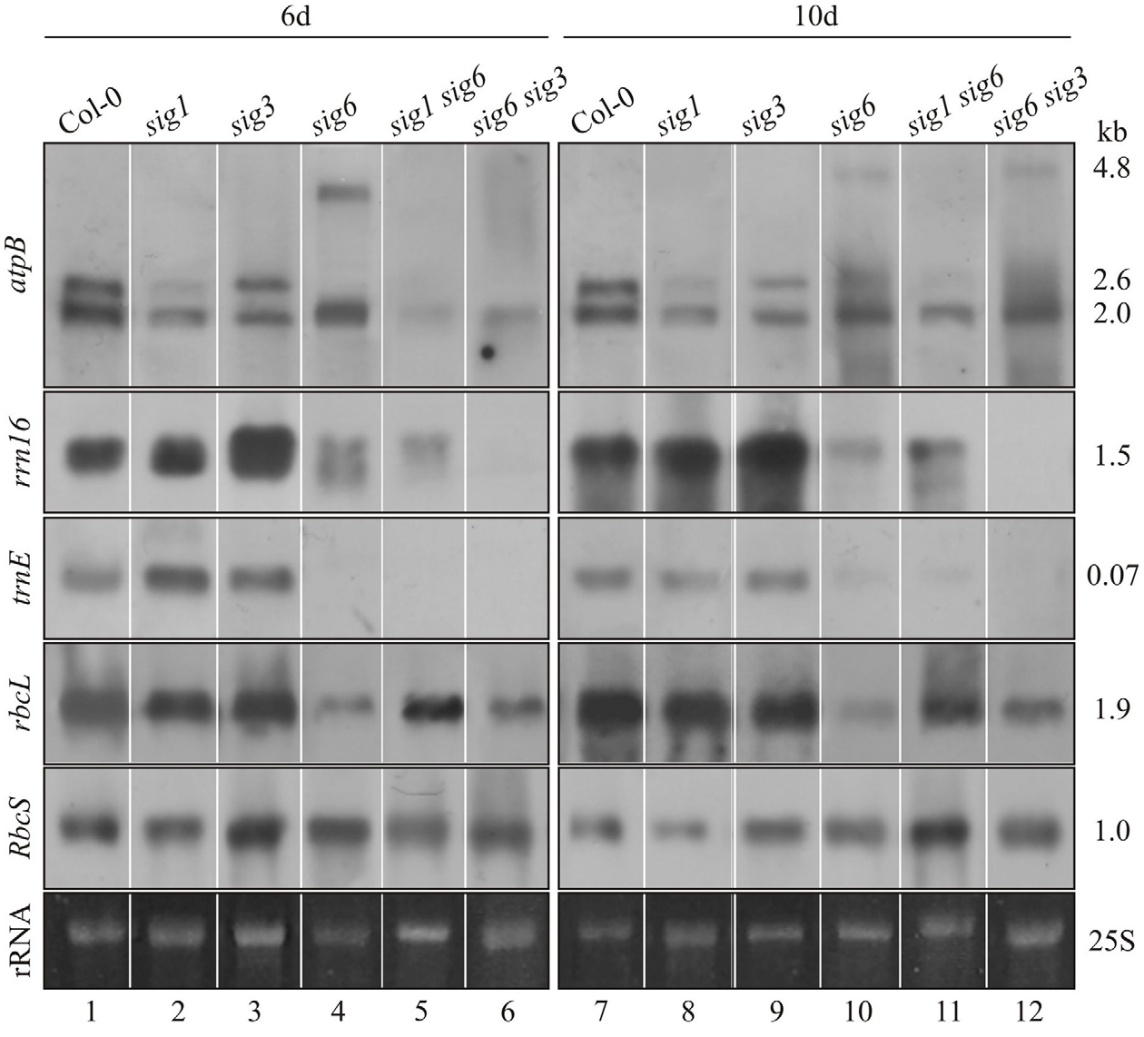
**Northern-blot analysis of plastid gene expression**. Col-0 wildtype as well as single and double knockout mutants were grown for 6 d (left panels) or 10 d (right panels). Total RNA (2 μg/lane) was fractionated, blotted and hybridized with the DIG-labeled RNA probes (*atpB, rrn16, trnE, rbcL*, and *RbcS*) as indicated the left margin. Ethidium bromide-stained loading controls (25S rRNA) are shown at the bottom. These experiments were carried out at least in triplicate with RNA from independent preparations. Right margin: transcript sizes (kb).

Using plastid RNA probes *rrn16, trnE*, and *rbcL* (Figure 2), no appreciable deviation from the Col-0 wildtype pattern (lane 1 and 7) was noticeable at 6 or 10 days for either the *sig3-2* or *sig1-2* mutant lines (lane 2, 3 and 8, 9), except for a relative increase in intensity of the *rrn16* signal at 6 d in the *sig3-2* line (lane 3). The *atpB* probe (top panel) revealed another difference, i.e., preferential loss of the 2.6 kb (PEP-dependent) transcript (Schweer et al., 2006) in the *sig1-2* line (lane 2 and 8). Unlike both the *sig3-2* and *sig1-2* lines, the *sig6-2* line shows overall less intense hybridization bands compared to wildtype (lane 4 and 10), although to a variable extent depending on the gene investigated. This is most evident for *atpB* (Loschelder et al., 2006), with a loss of the 2.6 kb (PEP-dependent) transcript and concomitant appearance of the 4.8 kb (NEP-dependent) “SOS” transcript (Schweer et al., 2006), particularly at the 6 day stage. Another gene showing dramatic down-regulation of transcript intensity in the *sig6* line is *trnE*, whereas the *rbcL* (Loschelder et al., 2006) and *rrn16* gene expression appears somewhat less affected and that of a nuclear (*RbcS*) control gene remains substantially unaffected (lane 4 and 10).

The double mutant lines *sig1 sig6* and *sig6 sig3* (lane 5, 6 and 11, 12) show a reduction in transcript intensities, which however only partly reflects the patterns of their parental single mutant lines. Following hybridization with the *atpB* probe (top panels), both double mutants show highly reduced signal intensity at the position of the 2.6 kb transcript (lane 2, 5, 6 and 8, 11, 12). In addition, also the 2.0 kb (NEP-dependent) signal is reduced at day 6 but reappears at day 10. The transcript detected with the *rrn16* probe (second panels) is present in reduced amounts in the *sig1 sig6* mutant (lane 5, 11) and is virtually absent in *sig6 sig3* (lane 6 and 12). The *trnE* signal (third panels) is not detectable in both double mutants at either time-point. In the *sig6 sig3* mutant (lane 6 and 12), the intensity of the *rbcL* hybridization signal (forth panels) resembles that of the parental *sig6-2* single mutant (lane 4, 10) and is diminished compared to the *sig3-2* signal (lane 3 and 9). The *sig1 sig6* signal (lane 5 and 11), however, is increased relative to that for *sig6-2* (lane 4 and 10) at both time points. It is comparable to that for *sig1-2* (lane 2) at 6 d (lane 5) but shows some relative decrease at 10 d (lane 11 vs. lane 8). Finally, the nuclear (*RbcS*) control reveals more uniform transcript intensity in all tested lines at 6 d (lane 1–6) than at 10 d (lane 7–12). At the latter time-point, the most notable effect is the relative increase in signal intensity for the sig1 sig6 line (lane 11) compared to the parental mutants and even the wildtype. This may reflect the known plastid to nuclear signaling (Woodson et al., 2012) in response to altered sigma-dependent chloroplast transcription (see “Discussion”).

### Sigma gene expression network in sigma single and double knockout lines

**Figure 3:**
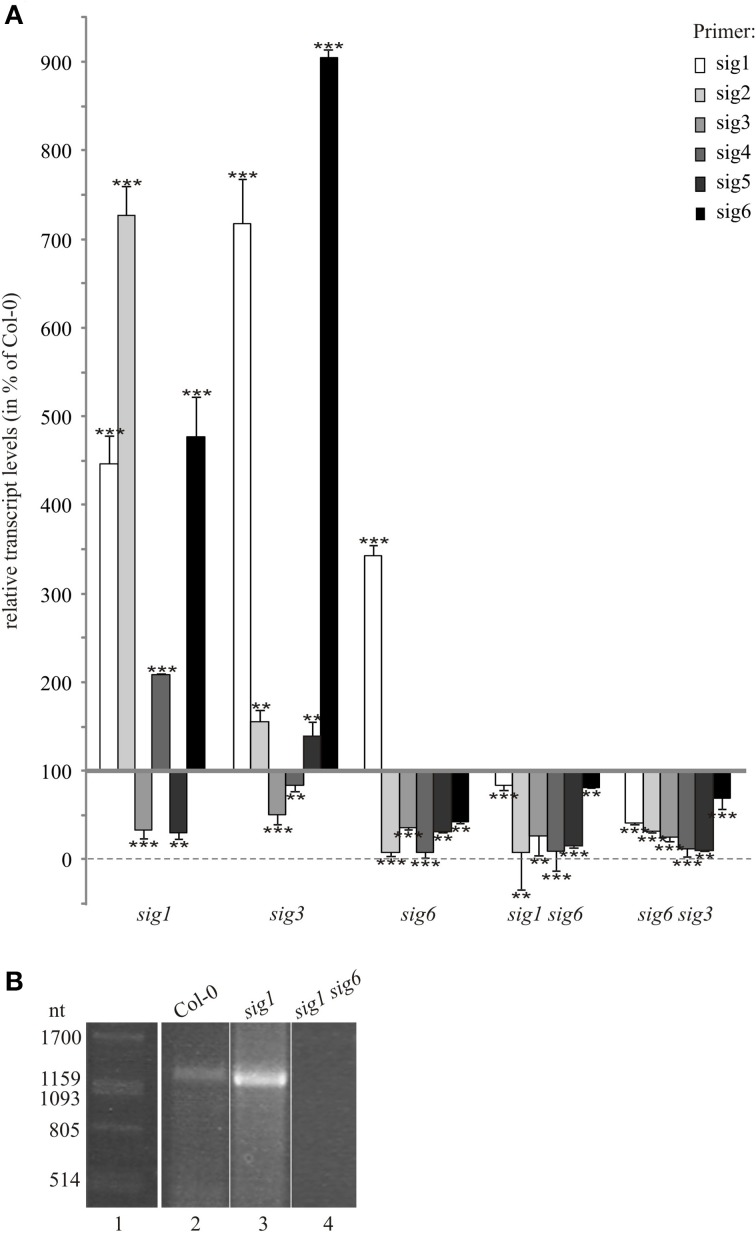
**Sigma transcripts in 10 d old knockout lines. (A)** RT-qPCR analysis. Transcript levels were calculated using the comparative threshold cycle method (Pfaffl, 2001) with wildtype plants as control and *Actin2* (At3g18780) as reference gene. Data are given as percentage of wildtype values (means ± SD of three biological replicates). Significance of deviation from Col-0 values was tested using Student's *t*-test and is indicated by two or tree asterisks (*P* < 0.05 and *P* < 0.001, respectively). **(B)** RT-PCR detection of a partial *SIG1* transcript spanning the distance between the start AUG and T-DNA insertion site. Total RNA was prepared from 10 d old seedlings of the Col-0 wildtype (lane 2), *sig1-2* single knockout (lane 3), and *sig1 sig6* double knockout line (lane 4). Following reverse transcription, the cDNA was amplified using the primer pair 5′-ATGGCTACTGCAGCTGTTATA-3′ and 5′-GAGTGTTGCACTTATA AGCTT-3′ for detection of partial *SIG1* transcripts. RNA markers (Promega) were used in lane 1 and their sizes (ntd) are given in the left margin.

To assess correlations between plastid target gene expression and sigma gene activity, transcript levels for all members of the sigma family were determined. To detect and quantitate these low-abundant transcripts, real-time qPCR rather than northern-blot hybridization was used. Whole-cell RNA preparations from 10 day-old homozygous single and double knockout lines as well as from the Col-0 wildtype were reverse-transcribed and subjected to real-time qPCR. Data were normalized to *Actin2* RNA expression levels (see “Materials and Methods”). As the RNA patterns of plastid target genes (Figure 2) indicated a close similarity of double mutant lines primarily with the *sig6-2* single mutant line, we tested these lines for correlation of target gene expression with sigma gene expression patterns themselves. As shown in Figure 3A for the *sig6-2* single mutant line, most sigma transcripts are down-regulated compared to wildtype, while the *SIG1* transcript seems to be strongly enhanced. In both double mutant lines, however, all six sigma transcripts including that of *SIG1* are reduced. Hence, except for *SIG1*, the expression phenotype of the sigma gene family in each double knockout substantially reflects that of the *sig6-2* parental line.

To test for possible similarity with other parental mutants, we also investigated *sig1-2* and *sig3-2* lines. In terms of their real-time transcript patterns (Figure 3A), these two lines each can be clearly set apart from *sig6-2* as well as from the two double mutants. In the *sig3-2* single knockout line, the only strong down-regulation is noticeable for transcripts detected by the *SIG3* primers, which is consistent with a major or full loss of *SIG3*-related transcripts in this mutant. While *SIG4* transcript levels are almost at wildtype level, those of *SIG2* and *SIG5* are moderately elevated and transcript levels of *SIG1* and *SIG6* are even strongly enhanced.

The *sig1-2* knockout line reveals transcript levels that are decreased in the case of *SIG3* and *SIG5*, but are increased for *SIG2, SIG4*, and *SIG6*. In addition, despite the T-DNA insertion interrupting the *SIG1* gene (Figure 1A) and the concomitant loss of a full-size *SIG1* transcript (Figure 1B), the RT-qPCR data for the *sig1-2* line (Figure 3A) suggested the existence of significant amounts of *SIG1*-related transcripts. The latter may represent partial (non-functional) transcripts upstream of the T-DNA insertion site. To test this, RT-PCR was carried out with primers that flank the distance from the ATG of the first exon to a site directly in front of the T-DNA insertion (Figure 3B). Both the wildtype (lane 2) and the *sig1-2* single mutant (lane 3) showed a PCR signal consistent with a transcript spanning the entire region defined by this primer pair. In contrast, such signal was not detectable in the double mutant line (*sig1 sig6*) (lane 4).

### Characterization of RNAi-modified sigma knockout lines

Attempts to further reduce the complexity of the sigma family were initially hampered by our inability to generate sigma triple-mutant lines, possibly because of their lethality. We therefore chose RNAi in combination with the existing single and double sigma mutants as an alternative strategy. “Combined” RNAi/knockout lines were created by transformation of the *sig6-2* and *sig6 sig3* mutants using constructs that contain the complete sequence for the unconserved sigma region (UCR) in pHELLSGATE12 (Wesley et al., 2001). Lines that could be stably maintained included those with the UCR sequences of *SIG2* or *SIG4* in the *sig6-2* single mutant background as well as those with the UCR sequences of *SIG1* or *SIG4* in the *sig6 sig3* double mutant background.

**Figure 4:**
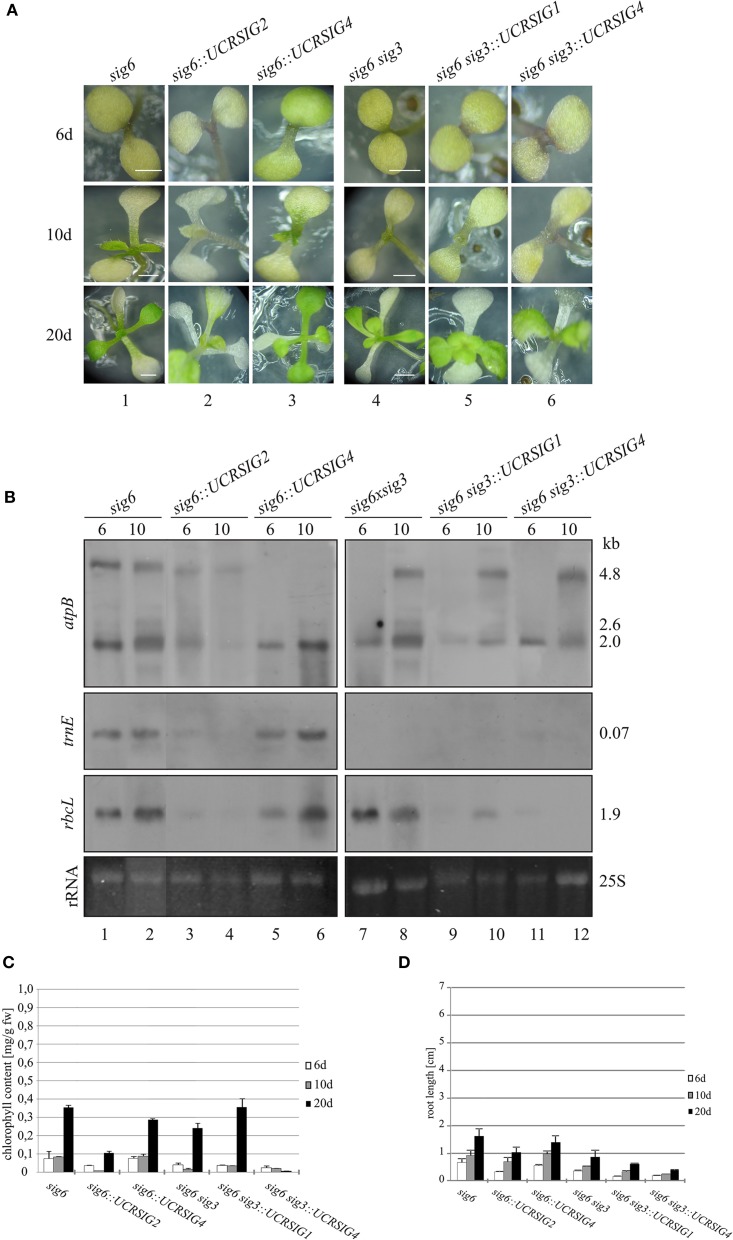
**“Combined” RNAi/knockout lines**. The single knockout line *sig6* and the double knockout line *sig6 sig3* each were transformed with the pHELLSGATE12 vector carrying *SIG1, SIG2*, or *SIG4* cDNA segments that represent the full-size UCR of sigma factors SIG1, SIG2, or SIG4, respectively (Table 1). **(A)** Phenotype of the RNAi/knockout lines and their parental lines during development (6, 10, and 20 d after sowing). Scale bars: 2 mm. **(B)** Northern blot analysis of plastid gene expression in RNAi/knockout lines and their parental lines. Total RNA (2 μg/lane) from 6 and 10 d old seedlings was fractionated, blotted and hybridized with *atpB, trnE*, or *rbcL* probes as indicated in the left margin. Ethidium bromide-stained loading controls (25S rRNA) are shown at the bottom. These experiments were carried out at least in triplicate with RNA from independent preparations. Right margin: transcript sizes (kb). **(C)** Chlorophyll content of seedlings and young plantlets 6 d, 10 d, or 20 d after sowing. Measurements involved 8 replicates of 10 samples each from three series of independently grown plant material. Weighed samples were ground in 80% (v/v) acetone and photometric chlorophyll determination at 663 and 645 nm was carried out. **(D)** Root lengths. Following growth of seedlings and young plantlets on vertically positioned Petri dishes, length measurements were carried out using ImageJ (http://imagej.nih.gov/ij). Values were means of three independent replicates using samples representing 20 seedlings or plantlets each.

At each time-point (6, 10, and 20 days after sowing) in Figure 4A, the combined RNAi/knockout line *sig6::UCRSIG4* (panels 3) has a pigment-deficient visible phenotype which resembles that of the parental *sig6* line (panels 1). The same is true for *sig6::UCRSIG2* (panels 2) as well as for combined lines *sig6 sig3::UCRSIG1 (*panels 5) and *sig6 sig3::UCRSIG4* (panels 6), and their parental double mutant line *sig6 sig3* (panels 4) (see also Figure 1C). The pigment-deficient visible appearance of all investigated RNAi/knockout lines is reflected by their chlorophyll content (Figure 4C). Likewise, root length measurements (Figure 4D) establish growth deficiency similar to that noticeable for the parental single (*sig6-2*) and double mutant lines (*sig6 sig3*) (see Figures 1D,E).

As shown in Figure 4B, transcripts of plastid target genes were found to be even more compromised than those of the (single and double mutant) parental lines (see also Figure 2), although differentially and to a variable extent. For instance, except for the loss of the 4.8 kb *atpB* transcript, the relative intensity of hybridization signals of *sig6::UCRSIG4* (panels 5 and 6) is comparable to that of *sig6-2* (panels 1 and 2) both at 6 d and 10 d, respectively. In contrast, *sig6::UCRSIG2* (panels 3 and 4) shows a weak but discernible signal (especially at 6 d) at the position of the 4.8 kb *atpB* transcript, while all other signals are virtually absent or highly reduced at both time-points in comparison with *sig6* (panels 1 and 2). This also includes the 2.0 kb band at the position of the NEP-dependent *atpB* transcript (Schweer et al., 2006), whereas the PEP-dependent 2.6 kb *atpB* transcript is absent both in *sig6* itself and in all *sig6*-derived lines. The transcript patterns of *sig6 sig3::UCRSIG1* (lanes 9 and 10) and *sig6 sig3::UCRSIG4* (panels 11 and 12) are similar to, but are more strongly affected than, that of their parental line *sig6 sig3* (panels 7 and 8). While all three lines show the 4.8 kb *atpB* transcript (at day 10 but not day 6), none of them reveals the 2.6 kb (PEP-dependent) *atpB* transcript and the 2.0 kb (NEP-dependent) transcript seems to be diminished in the combined RNAi/double knockout lines (panels 9–12). The *trnE* transcript is absent in all three lines (panels 7–12), and the *rbcL* transcript is highly reduced in the “combined” lines (lanes 9–12) compared to the parental double knockout (panels 7 and 8).

**Figure 5:**
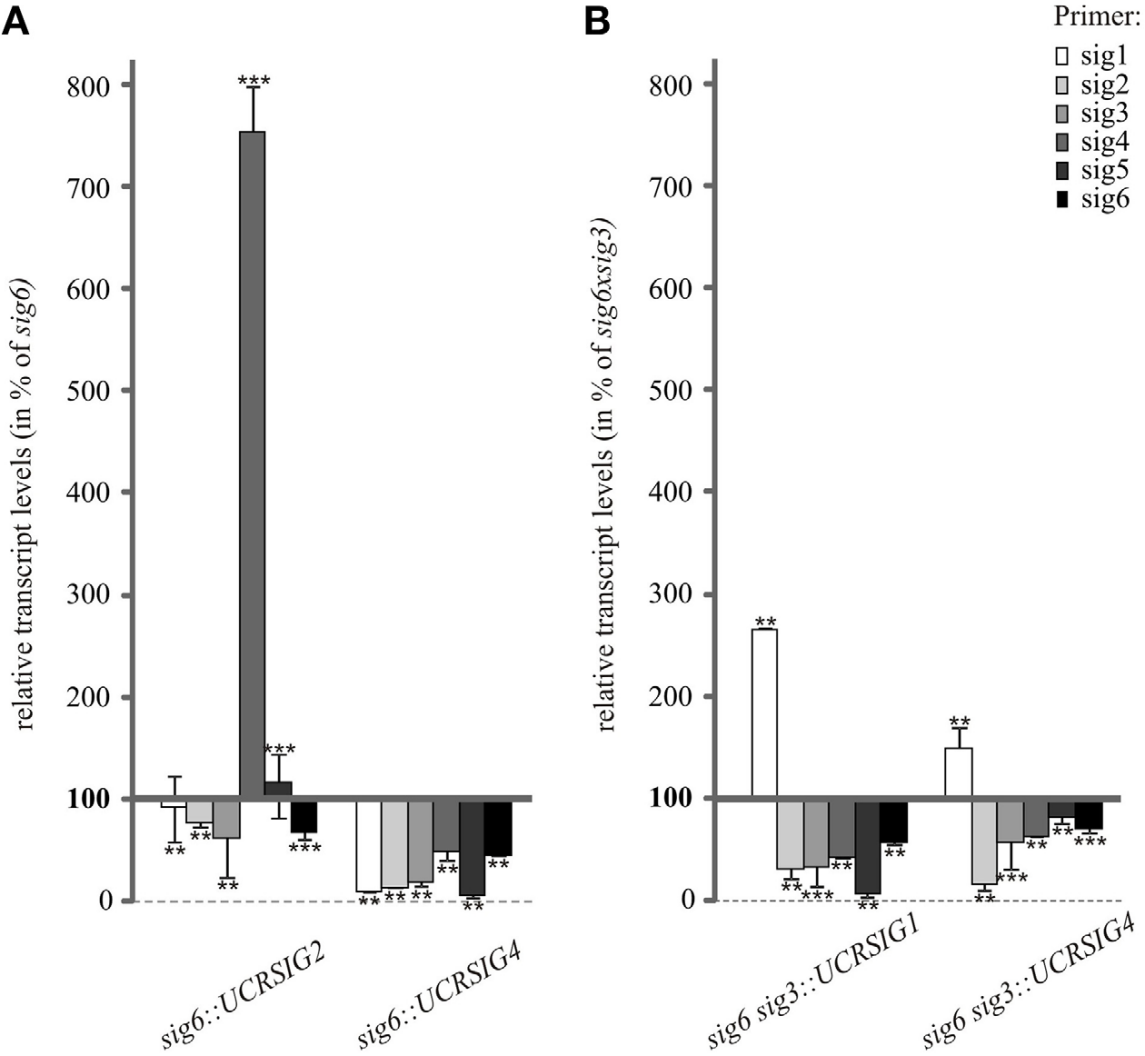
**RT-qPCR analysis of sigma transcript levels in 10 d old RNAi/knockout lines compared to their parental lines *sig6* (A) and *sig6 sig3* (B)**. Data are percent values of those of the parental mutant lines (set to 100%) and were normalized using *Actin2* as a reference gene. Data are means ± SD (*n* = 3). Asterisks indicate statistically significant differences compared to wildtype as tested by Student's *t*-test (*P* < 0.05).

The same RNAi/knockout lines were also tested for their sigma transcript patterns in comparison with those of the *sig6* and *sig6 sig3* parental lines (Figure 5). In the case of the *sig6::UCRSIG4* line, the steady-state transcript concentrations of all sigma genes are further reduced compared to those of *sig6* itself. In contrast, *sig6::UCRSIG2* shows a more diverse pattern, with a moderate further reduction of the *SIG2, SIG3*, and *SIG6* transcripts compared to the *sig6-2* line, substantially unchanged levels of the *SIG1* and *SIG5* transcripts, and strongly increased concentration of the *SIG4* transcript (Figure 5A). The RNAi/double knockout lines *sig6 sig3::UCRSIG1* and *sig6 sig3::UCRSIG4* (Figure 5B) both reveal moderate to strong further reduction of the *SIG2*–*SIG6* transcripts but enhanced levels of the *SIG1* transcript compared to the parental *sig6 sig3* line. Their transcript patterns thus seem more similar to one another than to those shown in Figure 5A.

## Discussion

This work was carried out to help reach a fuller understanding of the plant sigma genes, i.e., the small nuclear gene family for chloroplast transcription factors resembling the bacterial sigma transcription initiation factors. We sought to analyze phenotypic and molecular consequences of altered patterns of sigma gene activity in *Arabidopsis thaliana*. Such changes can be “homeostatic,” i.e., balanced and compensatory, which can be anticipated during developmental and/or physiological transitions. In more extreme situations, however, the sigma network can be thought to become “disrupted,” i.e., (irreversibly) imbalanced and rendered non-functional, as might be expected in lethal or heavily compromised mutants and/or under strong environmental stress. To narrow down the limits of homeostatic vs. imbalanced states of the sigma gene family, we have chosen strategies to differentially affect the sigma network, including single and double sigma mutants in combination with RNAi.

The parental single mutants, *sig1-2, sig3-2*, and *sig6-2*, were chosen for reasons of their specific phenotypic characteristics. *SIG6* single mutant lines reveal a clear-cut chlorophyll-deficient and developmental-stage-specific (seedling) phenotype, indicating a specialized and/or functionally dominating role of this factor (Ishizaki et al., 2005; Loschelder et al., 2006; Schweer et al., 2006, 2009). In contrast, *sig3* lines do not show pronounced pigment deficiency and the corresponding factor SIG3 has been assigned a functionally redundant role, perhaps as a possible safeguard in case of loss of other sigma factor(s) (Schweer, 2010; Lerbs-Mache, 2011). Although *sig1* knockout mutants of Arabidopsis have not yet been presented in terms of their gene expression characteristics, such mutants are of considerable interest in view of recent findings that sigma factor 1 (SIG1) is subject to phosphorylation control (Shimizu et al., 2010), as is known for sigma factor 6 (Schweer et al., 2010a,b; Türkeri et al., 2012).

To reduce the number of functional sigma genes, the *sig6-2* knockout line (Loschelder et al., 2006; Schweer et al., 2006, 2009) was crossed with either of two other single mutant lines, *sig1-2* and *sig3-2*, giving rise to the double mutants sig*1 sig6* and *sig6 sig3*. The latter reveal a growth-retarded and highly chlorophyll-deficient phenotype at seedling stage, even exceeding that of the parental *sig6-2* knockout. Plastid target gene expression at RNA level was strongly compromised in both double mutants. Assessment of sigma gene expression itself using RT-qPCR showed both losses but also increases in transcript frequency for individual members of the gene family in the single mutants. In contrast, however, a global decrease of sigma transcripts was noticeable in the double mutants. Hence, due to functional redundancy and compensation of sigma family members, a homeostatic balance seems still to prevail in the single knockout lines, while the balance may be strongly shifted or completely lost in the double mutants.

Attempts to further reduce the complexity of the sigma family by construction of triple mutants were unsuccessful, likely because of lethality of the progeny from these crosses. As an alternative, we therefore combined knockout mutant with RNAi technology. Using various sigma-specific unconserved regions (UCRs) in the pHELLSGATE12 RNAi vector, single (*sig6*) and double (*sig6 sig3*) mutant lines were transformed by constructs based on this vector. Resulting progeny lines generated by transformation of *sig6* showed a chlorophyll-deficient phenotype similar to or even stronger than that of the parental knockout line. Those generated from *sig6 sig3* did not reveal a further enhanced phenotype compared to the parental double knockout line, suggesting that a “basal” (minimal) state which cannot be further reduced without loss of viability may have been reached already in the latter.

**Figure 6:**
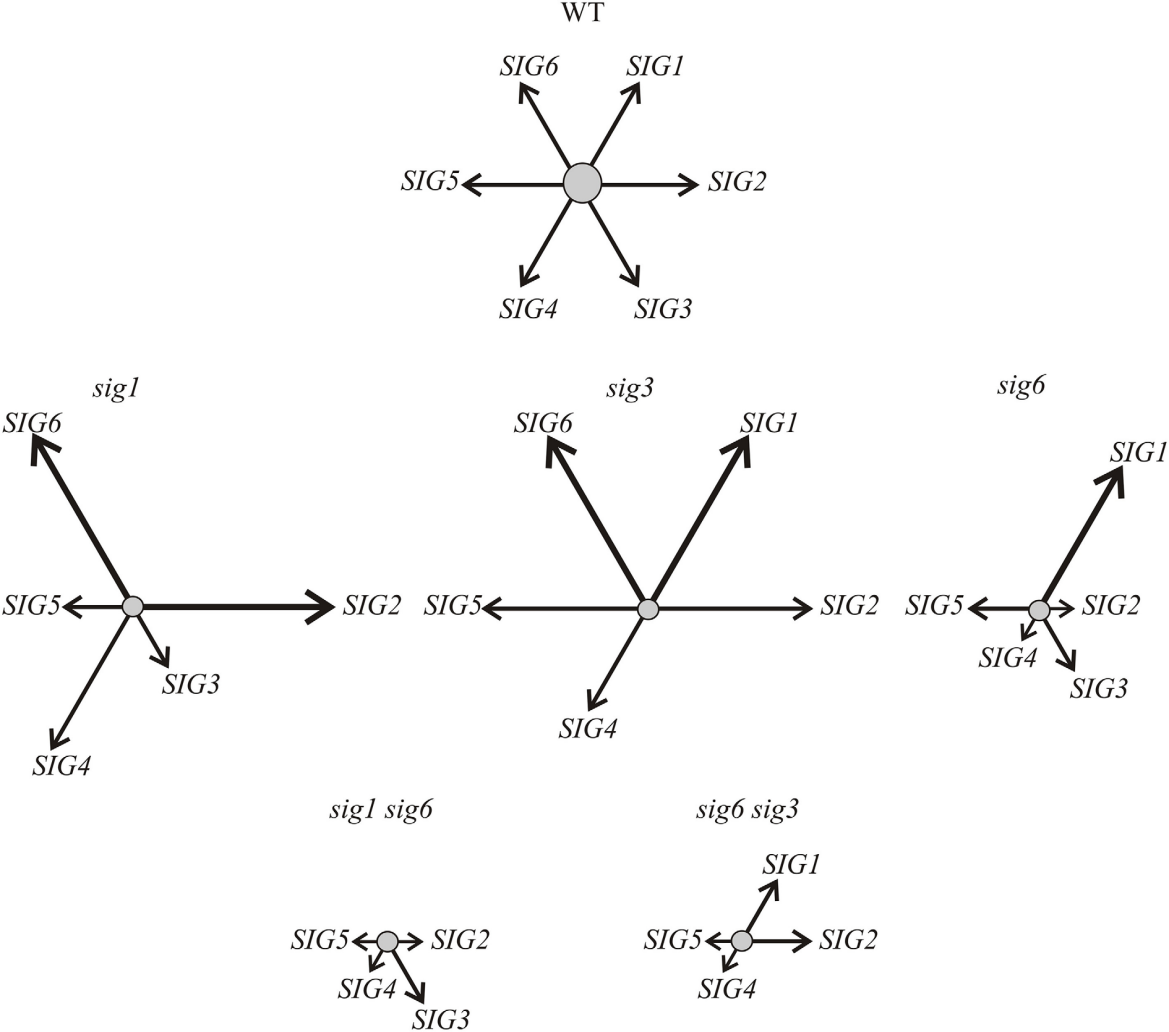
**Scheme symbolizing the transcript pattern of the sigma family in Arabidopsis mutant lines as revealed by RT-qPCR (Figure 3A)**. For simplicity, the balanced network of sigma factor transcripts (*SIG1*—*SIG6*) in the wildtype is indicated by equal length and strength of the arrows arranged in a hexagonal pattern **(top)**. As indicated by the altered length and strength of arrows, the contribution of individual sigma transcripts varies considerably in the single **(middle portion)** and double mutant lines **(bottom)**. Compensation of a completely lacking and/or non-functional transcript by overexpression of different member(s) of the gene family is noticeable in the single mutants (shifted, but probably still balanced, network). In the double mutants, this “valve” may no longer be available, as is suggested by the down-regulation of all remaining transcripts (imbalanced scrambled network). It should be noted, however, that the majority of the “combined” RNAi/knockout lines (Figure 5) still reveal increased (compensatory?) transcript levels of a single sigma gene (not shown here), suggesting that a (still viable) “basal state” may not have been reached yet in the double knockout mutants **(bottom row)**.

An argument against this notion, however, comes from results of the target gene expression studies, showing that the *rbcL* transcript is readily detectable in *sig6 sig3* but is highly reduced or absent in the “combined” RNAi/double knockout lines (Figure 4B). Furthermore, the RT-qPCR analysis (Figure 5B) shows enhanced transcript levels of the *SIG1* transcript in *sig6 sig3::UCRSIG1* and *sig6 sig3::UCRSIG4* as compared to the parental line *sig6 sig3*, which may reflect a still balanced functional state of the sigma gene family in these lines. In any case, it is notable that down-regulation of the expression of a single sigma gene can both negatively or positively affect that of another family member. For instance, the expression of *SIG1* and *SIG6* seems to be regulated in an opposite manner in single knockout lines (Figure 6), indicating functional redundancy and mutual compensation.

A perhaps unexpected finding is the partial or even dramatic loss of the 2.0 kb (NEP-dependent) *atpB* transcript (Schweer et al., 2006) in all RNAi/knockout lines (Figure 4B). Sigma factor SIG6 was previously implicated in retrograde signaling from the chloroplast to nucleus (Woodson et al., 2012), i.e., a mechanism that can affect the expression of nuclear genes in response to altered sigma factor function and chloroplast transcription (Pfannschmidt, 2010). It can be envisaged that the nuclear-encoded plastid polymerase might be regulated via this route, which in turn could explain the loss of the 2.0 kb NEP-dependent *atpB* transcript (Hanaoka et al., 2005).

Concerted regulation of both the PEP and NEP transcription systems via functional alterations of one or several sigma factors can be considered as efficient and flexible mechanism to achieve interorganellar integration. For instance, it might be interesting to investigate if the increased *RbcS* transcript level seen in the sig1 sig6 double mutant (Figure 2) is primarily due to an altered sigma network in this mutant and/or involves NEP-dependent regulation. Clearly, differential and compensatory expression of sigma genes as studied here is only one of several control levels. Posttranslational modification such as phosphorylation (Schweer et al., 2010a,b; Shimizu et al., 2010) as well as interactions of sigma facors with other regulatory proteins are equally important (Morikawa et al., 2002; Chi et al., 2010), as is the topology of the plastid transcriptome (Yagi et al., 2012; Zhelyazkova et al., 2012). In any case, our current work points to a causal relationship between the expression status of the sigma gene family and responses at the level of target gene expression.

Finally, it should be recalled that all RNAi lines described here were generated using the “constitutive” pHELLSGATE12 silencing vector (Wesley et al., 2001). A somewhat similar picture also emerges from initial recent work with chemically inducible dexamethasone (DEX)-responsive) RNAi lines based on the pOpOff2 vector (Wielopolska et al., 2005), providing proof of principle for DEX-responses that are visible at both target gene and sigma expression patterns (data not shown). Usage of such inducible “knock-down” system, also including, e.g., virus-induced gene silencing (VIGS) (Ratcliff et al., 1997), can be expected to open up new avenues in studies on temporal and spatial activities of the gene-containing plant cell organelles. This way, it should become possible to successfully analyze stages throughout the entire Arabidopsis life cycle.

## Author contributions

Sylvia Bock carried out planning, performance and presentation of most experiments. Jennifer Ortelt assisted in all aspects of experimental analyses and manuscript preparation. Gerhard Link provided advice and assistance throughout this work.

### Conflict of interest statement

The authors declare that the research was conducted in the absence of any commercial or financial relationships that could be construed as a potential conflict of interest.
